# Immunological Mechanisms in the Pathophysiology of Non-Alcoholic Steatohepatitis

**DOI:** 10.3390/ijms141019867

**Published:** 2013-10-01

**Authors:** Luisa Vonghia, Peter Michielsen, Sven Francque

**Affiliations:** 1 Department of Gastroenterology and Hepatology, University Hospital Antwerp, Wilrijkstraat 10, Edegem 2650, Belgium; E-Mails: peter.michielsen@uza.be (P.M.); sven.francque@uza.be (S.F.); 2 Department of Basic Medical Sciences, Neuroscience and Sensory Organs, University of Bari, Policlinico, Piazza Giulio Cesare, Bari 70100, Italy

**Keywords:** non-alcoholic steatohepatitis (NASH), innate immunity, adaptive immunity, adipokines

## Abstract

Non-alcoholic steatohepatitis (NASH) is characterized by the presence of steatosis, inflammation and hepatocyte injury and constitutes hepatic manifestation of the metabolic syndrome. The pathogenesis of NASH is complex and implicates cross-talk between different metabolically active sites, such as liver and adipose tissue. Obesity is considered a chronic low-grade inflammatory state and the liver has been recognized as being an “immunological organ”. The complex role of the immune system in the pathogenesis of NASH is currently raising great interest, also in view of the possible therapeutic potential of immunotherapy in NASH. This review focuses on the disturbances of the cells constituting the innate and adaptive immune system in the liver and in adipose tissue.

## 1. Introduction

Non-alcoholic fatty liver disease (NAFLD) is characterized by evidence of hepatic steatosis, in the absence of causes for secondary hepatic fat accumulation. The presence of steatosis and inflammation with hepatocyte injury (ballooning) defines non-alcoholic steatohepatitis (NASH), which may or may not be accompanied by fibrosis [1]. NAFLD constitutes a major health concern with the increasing incidence of obesity and diabetes in many Western countries. In the general European population the prevalence of NAFLD has been estimated between 2% and 44% (including obese paediatric populations) and between 42.6% and 69.5% in patients affected by type 2 diabetes [2]. In the USA 80 million adults have been estimated to be affected by NAFLD [3] ranging from around 31% in an unselected adult population (with higher prevalence in the Hispanic subgroup) [4,5], to 74% among diabetic subjects [6]. Moreover the prevalence of NAFLD has been progressively increasing in the Asian community to the current rates of 15%–30% [7,8].

NAFLD constitutes one of the three major causes of cirrhosis and liver transplantation, given the possible evolutive course of this disease, and can also be associated with the occurrence of a hepatocellular carcinoma. Intriguingly, hepatocellular carcinoma can occur in non-cirrhotic patients [9].

Moreover a close relationship has been highlighted between NAFLD and NASH and metabolic syndrome, associating visceral overweight, dyslipidaemia, hyperinsulinaemia or diabetes, and arterial hypertension [10]; furthermore NAFLD and NASH are currently considered as hepatic manifestations of the metabolic syndrome [11]. The strict link between NAFLD/NASH and insulin resistance is well established and it is highlighted by the implication of insulin signalling in the mechanisms that lead at different levels to the onset and progression of this disease. A key role in the development of insulin resistance is played by altered lipid metabolism that generates lipid intermediates, which in turn are able to activate different kinases, such as the mammalian target of rapamycin (mTOR), the inhibitor of κB-kinase (IKK), the c-Jun *N*-terminal kinase (JNK) and the novel protein kinase C (nPKC) [12]. The activation of these kinases has a negative feedback on proximal insulin signalling, contributing to insulin resistance and to a hyperinsulinemic state that further increases *de novo* liponeogenesis, hepatic lipid accumulation and disease progression [13].

The pathogenesis of NASH is complex and implicates cross-talk between different metabolically active sites. The initial “two hits” hypothesis described insulin resistance as “first hit” that leads to hepatic steatosis and is followed by a “second hit” driven by oxidative stress, which in turn leads to the development of steatohepatitis and fibrosis [14]. This model has been expanded in a “multiple parallel hits” hypothesis in which a number of different processes may contribute to liver inflammation. A crucial role is played by inflammatory mediators, especially those deriving from adipose tissue and the gut, which are involved in the cascade of inflammation, fibrosis and eventually tumorigenesis. In this setting, endoplasmic reticulum stress, cytokines and adipokines as well as immunity are emerging drivers of the key features of NASH [15]. Moreover the liver itself displays immune properties, and can be viewed as an “immunological organ” [16].

**Figure 1:**
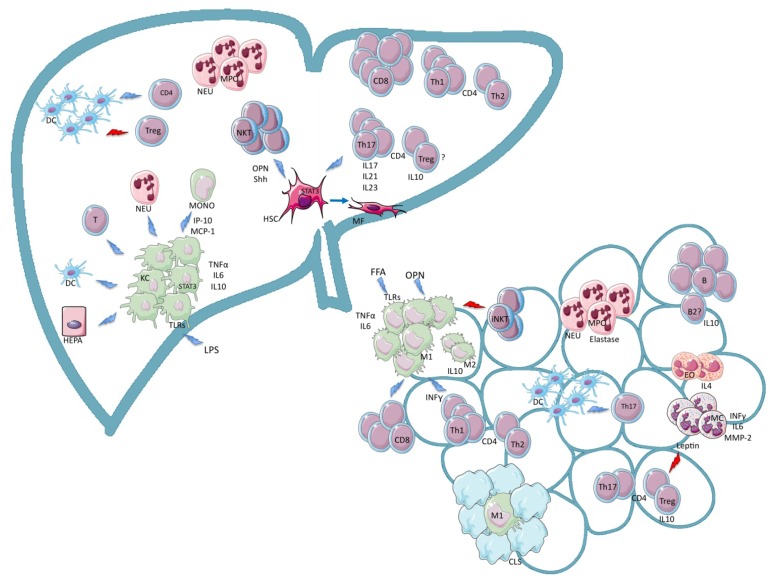
Overview of the immune pathways implicated in the pathogenesis of non-alcoholic steatohepatitis (NASH). DC: dendritic cells; Treg: T regulatory cell; NKT: natural killer T cells; NEU: neutrophil; MONO: monocyte; HEPA: hepatocyte; HSC: hepatic stellate cell; MF: myofibroblast; KC: Kupffer cell; EO: eosinophil; T: T lympohcyte; B: B lymphocyte; M: macrophage; CLS: crown like stucture; OPN: osteopontin; Shh: sonic hedgehog; LPS: lipopolysaccaride; FFA: free fatty acids; IP-10: interferon γ-induced protein-10; MCP-1: monocyte chemotactic protein-1; MMP-2: matrix metalloproteinase-2; MPO: myeloperoxidase; TLR: toll-like receptor; STAT3: signal transducer and activator of transcription 3; IL: interleukin. Blue lightning bolts indicate stimulation and red lightning bolts indicate inhibition. See text for details.

Many efforts have been undertaken to understand the role of the immune system in the pathogenesis of NASH, also in view of its potential therapeutic relevance. This review will focus on the disturbances of the cells constituting the innate and adaptive immune system in the liver and in the adipose tissue in NASH (Figure 1).

## 2. Innate Immunity

The innate immune system constitutes the first line of defence against invading pathogens. It comprises the cells and mechanisms that defend the host from infection by other organisms in a non-specific manner. The cells of the innate system recognize pathogens and provide a rapid but generic response. They are involved in recruiting immune cells to sites of infection and are able to activate the specific response of the adaptive immune system [17].

### 2.1. Macrophages/Kupffer Cells

Kupffer cells (KC) constitute the largest population of tissue resident macrophages. They derive from circulating monocytes that localize in the liver, where they are resident in the sinusoidal space, especially in the periportal area, where they clear endotoxins, debris and microorganisms. Under steady state conditions, KC can inhibit dendritic cell (DC)-induced antigen-specific T cell activation and can promote the suppressive activity of T regulatory cells (T regs) [18]. Upon activation by bacterial antigens, such as lipopolysaccaride (LPS), KC modulate the differentiation and activation of various immune cells, including DC, T lymphocytes and neutrophils. KC can also directly interact with hepatocytes, passing through the space of Disse [16]. Moreover, KC can contribute to liver injury through the production of pro-inflammatory cytokines, complement activation and reactive oxygen species (ROS) production [19].

KC are the first responding cells to hepatocyte injuries, leading to tumour necrosis factor-α (TNF-α) production, chemokine induction, and monocyte recruitment. In murine diet-induced NASH model, the early phase of NASH development is characterised by increase of TNF-α-producing KC, which in turn induce, via the production of interferon γ-induced protein-10 (IP-10) and monocyte chemotactic protein-1 (MCP-1), a later infiltration of pro-inflammatory CD11b^int^ Ly6C^hi^ monocytes. On the contrary, the depletion of KC reduced the incidence of liver injury, steatosis, and pro-inflammatory monocyte infiltration [20]. Moreover KC ablation can lead to less severe steatosis by blunting IL1β and nuclear factor (NF) κB suppression of peroxisome proliferator-activated receptor (PPAR)-α [21].

In a paediatric population, CD163+ cells accumulated in liver biopsies of NASH patients displaying severe disease. Moreover the entity of CD163+ infiltration correlated with the amount of steatosis and with the severity of disease [22].

KC are implicated in the onset of steatohepatitis also via toll-like receptor (TLR) signalling. TLR-9 stimulates the KC release of interleukin (IL) 1β, which is implicated in hepatocyte lipid accumulation, cell death and in fibrogenesis [23]. KC are sensitive to gut-derived endotoxin which also act through TLR-2 and TLR-4 [24]. LPS-mediated TLR-4 activation and induction of KC activity appeared to be important in the development and progression of NASH both in preclinical and clinical studies [25–27]. However, in methionine-choline deficient diet (MCD)-induced steatohepatitis, TLR-2 deficiency results in increased liver injury suggesting a protective role for TLR-2-mediated signals in liver injury [28].

KC are able to both secrete and respond to pro-inflammatory cytokines such as IL6 and also to anti-inflammatory cytokines such as IL10. An important role in the balance between pro- and anti-inflammatory responses is played by the signal transducer and activator of transcription 3 (STAT3), which after transient activation favours a pro-inflammatory response, while after prolonged activation drives an anti-inflammatory response [29]. Furthermore, IL6 is a key factor in the onset and progression of NASH and in the development of insulin resistance [30], while a relative deficiency of IL10 can be associated with NASH [27]. The role of myeloid STAT3 in hepatocellular damage is, however, still controversial, as STAT3 is able to blunt the expression of both pro-inflammatory cytokines and of hepatoprotective cytokines [29].

Recently, the role of macrophage infiltration and function in the adipose tissue was highlighted. It is well established that the behaviour of macrophages is heterogeneous, depending on the different environmental settings. Their activation ranges along a continuum between two separate polarization states: the “classically activated” pro-inflammatory M1 and the “alternatively activated” anti-inflammatory M2 states. M1 polarized macrophages are induced by pro-inflammatory mediators, such as LPS or interferon-γ (INF-γ), and, in turn, lead to the secretion of pro-inflammatory cytokines, as TNF-α, IL6, IL12, and to inducible nitric oxide synthase (iNOS) activation. These cells often aggregate around necrotic adipocytes forming characteristic “crown like” structures [31]. M2 polarized macrophages can be induced by various stimuli, mostly IL4 and IL13 [32]. However, M2 polarization can also occur in the absence of IL4 or IL13. IL 4-driven polarization is associated with lipid oxidative metabolism via the PPAR-γ pathway [33].

M2 polarized macrophages are characterized by the production of anti-inflammatory cytokines, as IL10 and IL1 decoy receptor, and the enhancement of arginase, an enzyme with i-NOS blocking properties [34]. This subset of cells is present in adipose tissue of lean mice, suggesting a potential beneficial function of these cells. Moreover M2 (F4/80+CD11c−CD206+) macrophages are decreased in obesity. These findings are in line with the protective role of IL10 in insulin resistance, balancing the effect of pro-inflammatory cytokines as TNF-α [35]. On the contrary, in diet, induced murine models of obesity macrophages switch to M1 profile. In fact, F4/80+CD11b+CD11c+ [32] cells as well as F4/80+CD11c+CD206− [36] cells, which are associated with the M1-polarization state, have been described as accumulating in adipose tissue and to overexpress inflammatory genes in a mice fed high fat diet (HFD). Surprisingly IL10 was also overexpressed after HFD, probably in relation to the recruitment of M2 macrophages into adipose tissue in response to HFD [36]. The obesity-related phenotypic switch from M2 to M1 polarization appears to be related to C-C chemokine receptor 2 (CCR2)-dependent monocyte recruitment rather than to the transformation of resident M2 macrophages [37]. The accumulation of M1 polarized macrophages can be influenced also by the interaction with other immune cells. For example the suppression of T cell activation can reduce pro-inflammatory adipose tissue M1 polarized F4/80+CD11b+CD11c+ macrophages of mice fed an obesogenic diet [38].

Moreover, in morbidly obese patients the recruitment of macrophages in the adipose tissue appears to be depot-specific. Macrophage recruitment has been shown to be enhanced in the omental and in the deep subcutaneous adipose tissue but not in the superficial adipose tissue of morbidly obese patients with NASH and/or fibro-inflammatory hepatic lesions. In addition, in this subset of morbidly obese patients, adipose tissue macrophage infiltration follows the increase of osteopontin (OPN), a cytokine highly secreted by macrophages, which is able to induce monocyte adhesion, migration and differentiation and phagocytosis, in the subcutaneous site and is reversible after surgery-induced weight loss [39]. Blunting macrophage accumulation, through osteopontin or monocyte chemoattractant protein-1/chemokine (C-C motif) ligand 2 (MCP-1/CCL2) inhibition, or ablation of specific subsets of macrophages induces an improvement of different aspects of NASH and metabolic syndrome, namely inflammatory activity, insulin resistance and hepatic fibrosis [30].

Finally in the adipose tissue of HFD-fed mice, macrophage activation can be induced via free fatty acid recognition by TLR-4 and TLR-2 [40], highlighting the role of TLR signalling also at this level.

### 2.2. Natural Killer T Cells (NKT)

NKT respond to non-peptidic antigens, as lipid and glycolipid antigens, presented by CD1d that can be expressed by hepatocytes and antigen presenting cells, such as macrophages, DC and B cells. NKT activation by IL12, which is released by DC and KC, results in Fas-mediated target cell lysis. Invariant NKT (iNKT) compose a unique highly conserved population which expresses invariant T cell receptors (TCRs) Vα24Jα18, paired with Vβ11 in humans and Vα14Jα18 coupled with TCR Vβ7, Vβ2 in mice [31]. Upon activation, NTK produce both Th1, pro-inflammatory/anti-fibrotic (INF-γ) and Th2, anti-inflammatory/pro-fibrotic (IL4 and IL13) cytokines, probably accounting for their immunoregulatory potential [16]. In addition to classical cytokines, NTK can also secrete OPN [41], which is implicated in liver injury progression [42] and the fetal morphogen, sonic hedgehog (Shh), which activates hepatic stellate cells (HSC) into collagen secreting myofibroblasts and amplifies the repair-associated inflammatory response [43]. NKT cells are generally depleted in liver steatosis [44–46], but are increased in NASH-related liver fibrosis [44,47]. In a mouse model of diet-induced NASH, the development of liver fibrosis was reduced in mice lacking all the NKT subsets [48] as well as in mice specifically depleted of invariant (iNKT) [49]. In addition NKT deficient mice fed a MCD diet, showed a blunted hedgehog (Hh) and OPN expression and a decrease in fibrogenic factors able to activate collagen gene expression in HSC. In the setting of human NASH, advanced fibosis correlated with increased hepatic levels of OPN and Hh and plasma OPN levels in comparison with early fibrosis [49].

iNTK appeared to play an important role also at the level of adipose tissue. iNKT are expanded in human [50] and murine [31] adipose tissue, where they represent a unique subset with distinct Th2 cell cytokine profile. In obesity, iNTK appeared to be reduced, in correlation with pro-inflammatory macrophage infiltration. Recent finding showed that iNKT-depleted mice fed HFD displayed increased weight gain, larger adipocytes, more liver steatosis and insulin resistance compared to the wild type mice. In contrast, adoptive transfer of iNKT or *in vivo* activation of iNKT via the lipid ligand α-galacotcylceramide, decreased fat accumulation, triglyceride and leptin (a pro-inflammatory adipokine) levels, liver steatosis and improved insulin sensitivity via anti-inflammatory cytokine production [31]. These data highlight the important role played by this subset of cells in liver injury progression, adipose tissue and metabolic regulation.

### 2.3. Neutrophil, Basophil, and Eosinophil Granulocytes, and Mast cells

Some studies have investigated the role of these cells in the pathophysiology of NASH, although it still needs to be better established. Neutrophils have been described to be implicated in the early phase of adipose tissue inflammation, since they can transiently infiltrate fat pads of mice fed HFD already within the first week of diet [51,52]. Moreover myeloperoxidase (MPO), an oxidant-generating neutrophil enzyme, has been suggested to be involved in prompting lipid peroxidation in steatotic livers, a process that favours the evolution from simple steatosis to steatohepatitis.

Additional evidence of neutrophil implication in the progression of NASH came from the observation that neutrophils and MPO-mediated oxidation products were increased in liver biopsies of NASH patients in comparison with simple statosis [53]. Furthermore low-density lipoprotein (LDL)-deficient mice fed HFD display a hepatic sequestration of MPO+ neutrophils and increased MPO activity. MPO deficiency decreases the hepatic neutrophil infiltration, TNF-α and IL6 expression, liver cholesterol accumulation, liver fibrosis and adipose tissue inflammation in response to HFD [54]. Moreover the ability of neutrophils to induce insulin resistance in mice fed HFD seems to be, at least in part, driven by elastase, a protease secreted by neutrophils which can promote inflammatory response. Deletion of neutrophil elastase in this experimental model leads to less tissue inflammation with lower adipose tissue neutrophil and macrophage content, together with improved glucose/insulin homeostasis [52].

Eosinophils constitute the major IL4 producing cells in murine adipose tissue. Eosinophil depletion favours the HFD induced development of impairment of glucose tolerance and insulin resistance, while helminth-induced adipose tissue eosinophilia is able to ameliorate glucose metabolism [55].

Based on epidemiological and clinical studies, an association between obesity and allergy has been postulated, therefore leptin was investigated as a possible mediator of this link. In fact, leptin receptor is expressed by human basophils and its activation strongly promotes basophil activation and degranulation, suggesting that leptin plays a crucial role in mediating the effect of adipocytes on inflammatory cells, including basophils [56].

Also mast cells have been found to be increased in the white adipose tissue of obese humans and mice [57], especially in the proximity of fibrotic depots and with a more prominent infiltration in abdominal fat *versus* subcutaneous fat [58,59]. Moreover, mast cell infiltration in the adipose tissue seems to correlate with a disturbed glucose metabolism, as well as with the presence of fibrosis, macrophage accumulation and endothelial cell inflammation [58]. After genetic mast cell-depletion or pharmacological stabilization, a reduction of body weight gain, inflammatory cytokines, chemokines and proteases was observed together with improvement of glucose metabolism and energy expenditure. In addition adoptive transfer experiments of cytokine-deficient mast cells showed a key role of IL6 and INF-γ produced by mast cells in developing diet-induced obesity and glucose intolerance [57]. Furthermore, in experimental murine models it has been shown that mast cells could play a role in the pathogenesis of liver fibrosis and may contribute to the degradation of fibrosis by synthesizing and secreting matrix metalloproteinase-2 (MMP-2) [60].

### 2.4. Dendritic Cells

DC are professional antigen presenting cells that can be located in extra-lymphoid tissues, including the liver, where they reside around the central veins and portal tracts. Upon activation they can migrate through the Space of Disse to the lymphatic ducts and portal tracts to reach extrahepatic lymph nodes. DC are implicated in the induction of central and peripheral immunological tolerance, in the regulation of the T cell immune response and they act as sentinel cells of innate immunity in the recognition of microbial pathogens. The specific function of DC is dependent on the heterogeneity of DC subsets and their functional plasticity [16].

Recently DC have been recognized to be mediators of non-infectious chronic inflammatory conditions. Liver fibrosis has been associated with a highly pro-inflammatory DC status [61], however other authors have described an accelerated resolution of liver fibrosis induced by DC recruitment [62]. A recent study utilizing a continuous *in vivo* DC depletion model highlighted that DC are able to limit NASH-related fibroinflammatory injury. DC appeared to be recruited in the early phases of NASH development by MCD diet, displayed phenotypic maturation (as shown by the expression of major histocompatibility complex class II (MHC II) and CD40, necessary for antigen presentation, and of the costimulatory molecules CD54, CD80 and CD86) and activation. When analyzing the different subsets of DC, the plasmocytoid DC (B220+) fraction was decreased while the CD11b+CD8a− myeloid fraction was expanded, whereas the fraction of CD11b−CD8a+ lymphoid DC decreased proportionately. Additionally, hepatic NASH DC showed an increased production of pro-inflammatory cytokines and *in vitro* induced proliferation of allogenic T cells and antigen-restricted CD4+ T cells, and down-regulated the expression of T regs. Depletion of DC in NASH markedly exacerbated intrahepatic fibro-inflammation and, accordingly, when DC were depleted during the recovery phase of the disease, a delayed resolution of the intrahepatic fibro-inflammation lesions was observed [63].

In adipose tissue, an accumulation of DC was described in mice (CD11c^hi^ F4/80^low^) and in humans (CD11c+CD1c+). In mice DC induced Th17 differentiation; in humans DC deriving from subcutaneous adipose tissue correlated with Body Mass Index (BMI) and with increased Th17 cells. When analyzing the morbidly obese patients, DC-related gene expression correlated with insulin resistance [64].

## 3. Adaptive Immunity

Adaptive immunity is characterized by antigenic specificity, diversity, immunologic memory, and self-nonself-recognition. This immune response is mediated by T and B lymphocytes and a variety of molecules that orchestrate cellular interactions [17].

### 3.1. T Lymphocytes

Although the total population of hepatic T lymphocytes (CD3+ lymphocytes) appears relatively stable in NASH, an imbalance of the different subtypes of CD3+ cells is observed in NASH. In particular an increased CD8+/CD4+ cell ratio has been described in the liver [63]. At the level of the visceral adipose tissue, an increase of CD3+ cells has been described in humans and in mice and CD3 mRNA correlated with BMI [65]. Accumulation of CD8+ and CD4+ cells was observed in adipose tissue inflammation [66] and the latter cell subtype showed a TCR repertoire bias, suggestive of antigen-driven T cell activation, expansion and infiltration [67]. CD4+ T cell transfer can negatively regulate weight gain, visceral adipose tissue mass, hyperglycaemia and cytokine increase (TNF-α and IL6) induced by HFD, predominantly through Th2 cells [67].

T helper cells are a sub-group of lymphocytes that play an important role in the immune system and in particular in the adaptive immunity. Through cytokine release, they are able to drive the activation of the other immune cells as they are implicated in the B cell antibody class switching, in the activation of the cytotoxic T cells and in maximizing the bactericidal activity of phagocytes such as macrophages. Depending on the cytokine environment, T helper cells can assume a pro-inflammatory phenotype (Th1), characterized by the release of INF-γ and transforming growth factor-β (TGF-β) or an anti-inflammatory phenotype (Th2), characterized by the release of IL4, IL5, IL13. The equilibrium between Th1 and Th2 is important in driving the immune response. An imbalance between a relative excess of pro-inflammatory cytokines and a relative deficiency of anti-inflammatory cytokines has been found in the context of NASH both in the liver [24] and in the visceral adipose tissue [67]. Accordingly, in a paediatric obese population, INF-γ expressing CD4+ cells were increased in the peripheral blood and correlated with insulinemia and clinical features of fatty liver disease [68].

Th1 enhancement can induce, via INF-γ, the infiltration of M1 polarized macrophages in the adipose tissue of obese mice, accompanied by increased expression of TNF-α and MCP-1 [69].

Cytotoxic T cells (CD8+) rapidly increase in fad pads during HFD [70], prior to macrophage infiltration, and express a highly activated phenotype characterized by the release of pro-inflammatory mediators, which are implicated in the recruitment and activation of macrophages in the adipose tissue. Immunological or genetic depletion of CD8+ cells reduces macrophage infiltration, adipose tissue release of proinflammatory mediators (such as IL1, IL6 and MCP-1), and insulin resistance [71]. These data highlight the role of CD8+ cells in initiating and propagating adipose tissue inflammation. Moreover the stimulating effect of CD8+ cells on macrophages, together with the loss of T regs in the adipose tissue of obesogenic models, that will be further discussed, may provide a positive feedback loop that could ultimately promote autoimmune phenomena in the context of obesity [72].

Other important subsets of T lymphocytes are the T regs and the CD4+ IL17-secreting Th17 cells. The balance between these subsets of cells is important in maintaining immune homeostasis, since this axis is skewed in many autoimmune, infectious and metabolic diseases [73].

### 3.2. T Regulatory Cells

T reg cells derive from CD4+ Th0 cells in the presence of TGF-β and constitutively express the CD25 (IL2 receptor α chain). In addition, they express CD62L (glucocorticoid induced tumour necrosis factor receptor), CTLA4 (cytotoxic T lymphocyte associated protein) and FOXP3 (forkhead/winged helix transcription factor), the latter being crucial for their function [74]. They are involved in the prevention of the proliferation of autoreactive cells, as in autoimmune hepatitis, as well as in the negative control of various immune responses, as viral hepatitis and hepatocellular carcinoma, and in promoting tolerance induction after organ transplantation [75]. In an HFD mouse model, a liver specific and reversible depletion of T regs was observed. T regs were able to blunt the HFD-induced pro-inflammatory milieu, as highlighted by the ability of T reg adoptive transfer to reduce the level of TNF-α expression and its downstream signalling. Moreover T regs showed a higher susceptibility to apoptosis related to oxidative stress in comparison to T effectors, while antioxidant treatment reduced hepatic inflammation and T reg apoptosis and restored the number of T regs. These data suggest that the link between steatosis-induced oxidative stress and T reg apoptosis could play a role in inducing hepatic inflammation. Moreover they provide a potential interventional strategy for NASH by modulating liver inflammation through a regulation of T reg number/function and apoptosis [75].

Contrary to these data, immunohistochemical evaluation of liver biopsies from NAFLD/NASH patients showed an increase of FOXP3+ cells in NASH patients with a more advanced disease. FOXP3 positivity was distributed both in the lobule and in the portal tracts and higher FOXP3+/CD3+ quota positively correlated with a the histologic severity of the disease. Therefore it could be postulated that T regs could be involved in the development of liver damage and that CD3+ cells could be diminished by T regs to decrease inflammation [76].

Furthermore, Tregs were investigated in the different sites of adipose tissue. FOXP3/CD4 expression was increased in the abdominal fat of 30 week old mice fed a normal diet, FOXP3 being expressed in more than half of the CD4+ cells, in comparison with lymphoid or non-lymphoid tissues, such as liver and subcutaneous fat. Low T reg expression was observed in both the abdominal and subcutaneous fat deposits at birth and progressively accumulated in the abdominal adipose tissue but not in the subcutaneous tissue over time. Moreover T regs were specifically reduced in the abdominal site in insulin-resistant models of obesity [67,77], with a mechanism related, at least in part, to the suppressive ability of leptin on T reg proliferation [78]. This dichotomy between subcutaneous and abdominal fat is in line with the well known association of the latter with insulin resistance [79]. In gain of function and loss of function experiments, the abdominal fat derived T regs indeed regulated the inflammatory state of adipose tissue and insulin resistance [77]. These cells have been identified as a distinct, tissue-specific subset of cells in which PPAR-γ seems to play a determinant role in T reg accumulation, phenotype and function in visceral adipose tissue [80]. When analysing human biopsy samples of obese patients, FOXP3 RNA was detected both in subcutaneous and in omental fat and it was expressed at a higher level in the subcutaneous district. Moreover a negative correlation between BMI and the FOXP3 to CD3 ratio in omental *versus* subcutaneous fat was reported in these patients [77]. Accordingly, investigating obese patients with and without insulin resistance, FOXP3 RNA levels in the visceral adipose tissue were lower only in obese patients without insulin resistance, while no difference was found when comparing insulin resistant obese patients and lean controls [81]. Additionally, to better understand the link between insulin resistance and T reg infiltration in the adipose tissue, T reg depletion and adoptive transfer were performed in an *ob/ob* mouse model. The former led to increased fasting blood glucose level, impaired insulin sensitivity and renal impairment, while the latter improved insulin resistance [81].

In type 2 diabetes the ratio between T regs and Th17 was decreased. T regs appeared to be more prone to cell death and their reduction was more pronounced in patients with microvascular rather than with macrovascular complications [82]. The regulation of the T reg/Th17 axis in these patients could be, at least in part, due to the action of IL6 and to its capability to interact with these cells either by binding the IL6 receptor (IL6-R) on different cell types or though a trans-signalling mechanism that involves the soluble sIL6-R [83].

### 3.3. Th17

Th17 cells are a subtype of T helper cells that are characterized by the secretion IL17 and which differentiation is specifically induced by the transcription nuclear factor retinoic acid receptor-related orphan receptor (ROR)-γt. Th17 are generated in the presence of TGF-β and IL6 and exert pro-inflammatory functions [84].

This subset of cells functionally opposes T reg mediated response and has reciprocal developmental pathways, having antithetical effects in the immune response. The T reg transcription factor FOXP3 has a direct inhibitory effect on the differentiation of Th17, binding the Th17 specific transcription factor ROR-γt. Moreover T regs may convert to Th17 in the context of pro-inflammatory stimuli, losing their suppressive function [85].

In the context of fatty liver disease, a higher number of Th17 cells has been described in an HFD mouse model of NALFD and in liver biopsies of NASH patients compared with controls. Accordingly, the Th17 related genes (ROR-γt, IL17, IL21, IL23) were upregulated in NASH patients *versus* controls. In addition neutralization of IL17 in the HFD diet fed mice decreased the LPS induced liver injury as indicated by reduced ALT and inflammatory infiltrate in the liver. *In vitro* tests showed that IL17 synergically contribute with the free fatty acids (FFA) to the development of steatosis via insulin signalling pathway interference [86].

Moreover IL17 and IL23, which are implicated in the Th17 pathway, appeared to be increased in obese patients and positively correlated with elevated levels of leptin, a pro-inflammatory and anorexigenic adipokine [87].

In line with these findings, a recent study showed that leptin deficient *(ob/ob)* or leptin-receptor deficient (*db/db*) mice displayed lower levels of IL17, in comparison to wild type (WT) mice, indicating an impairment of the IL17 pathway in conditions of leptin downregulation. Moreover IL17 pathway resulted in being enhanced, in a dose dependent manner, by leptin. Indeed, increasing doses of leptin were able to raise the number of splenic Th17 as well as the production of IL17 and of the Th17-specific transcription nuclear factor ROR-γt in *ob/ob* leptin deficient mice [88].

Contrary to these findings, Th17 were reduced in mice fed HFD at the level of visceral adipose tissue [67] and IL17 acted as a negative regulator of adipogenesis and glucose metabolism in mice, and delayed the development of obesity [89].

The IL17 pathway appears to be implicated also in the pathogenesis of liver fibrosis, which plays a key role in the progression of liver disease. In hepatotoxic and cholestatic mouse models, liver injury enhanced IL17 signalling, as revealed by the increase of IL17 and its receptor and by the consequent activation of inflammatory and liver resident cells. In fact, IL17 induced an increased production of IL6, IL1, TNF-α and TGF-β1 by inflammatory cells as well as an increased deposition of collagen type 1 by HSC, via signal transducer and activator of transcription 3 (STAT3) activation [90].

A link between obesity-related disturbance of the Th17 pathway and autoimmunity has been described. In diet-induced obese mice, the obesity-related enhancement of the Th17 pathway has been shown to be correlated to a more pronounced experimentally induced autoimmune disease [91] and leptin promoted the Th17 response in lupus-prone mice, indicating a connection between metabolism, nutrition and susceptibility to autoimmunity [88].

### 3.4. B Lymphocytes

B lymphocytes constitute around 6% of intrahepatic cells [16]. Moreover they rapidly increase in serum and adipose tissue of mice fed HFD, and seem to be implicated in insulin resistance. Namely, B-cell-deficient mice fed HFD show a lower insulin resistance than controls. Accordingly, adoptive transfer of B cells or IgG isolated from mice fed HFD into B-cell-deficient mice determines the onset of insulin resistance. Moreover patients with insulin resistance display a distinct IgG profile compared to subjects without insulin resistance [92].

In addition, an increase of the serum level of B-cell-activating factor (BAFF) has been described in human NASH. Preclinical studies showed that BAFF receptor deficient mice display an improvement in HFD-induced obesity and insulin resistance accompanied by a reduction of B cells, serum IgG levels and visceral adipose tissue inflammation. Moreover BAFF was found to be able to downregulate steatogenesis genes and to enhance steatosis in hepatocytes through BAFF-R, indicating a protective role of BAFF in hepatic steatosis by regulating lipid metabolism in the liver [93].

Intriguingly, mouse and human omenta are sources of B cells during ontogeny, especially of the B1 subset, that infiltrate the omentum and the peritoneal cavity in adults. B1 cells peculiarly respond to pathogens in a faster manner and with a less diverse but more polyreactive antibody repertoire than “conventional” B2 cells and can express IL10. These IL10 producing B cells, also called “B regs”, are implicated in modulating autoimmune manifestations [94] and could potentially play a role in the obesity-related susceptibility to autoimmunity [72]. B2 cells could also be altered in obesity as suggested by the observation of a lower antibody response to tetanus toxin immunization in overweight children and mice [95,96].

## 4. Adipokines and Soluble Mediators

A relevant role in the frame of the “multiple parallel hits hypothesis” is played by the balance of adipose tissue derived mediators, such as adiponectin and leptin [15]. Moreover the role of ghrelin, visfatin and resistin has been investigated [97,98] (Table 1).

Adiponectin is produced mainly by the adipose tissue but also to a lesser extent by other organs such as bone marrow, fetal tissue, cardiomyocytes and hepatic endotelial cells. Principally, adiponectin has insulin-sensitizing and anti-inflammatory properties. Plasma adiponectin levels inversely correlate with BMI, where lower levels have been found in subjects with visceral fat accumulation and in diabetic patients *versus* non-diabetic ones. Accordingly, hypo-adiponectinemia has been found to be associated with metabolic syndrome, type 2 diabetes, hypertension, atherosclerosis and NASH [99].

The anti-inflammatory action of adiponectin in the liver could be driven by the inhibition of the production of pro-inflammatory cytokine, such as TNF-α, the stimulation of the release of IL10 by KC [100] and the direct suppression of macrophage function [101]. Moreover adiponectin is able to attenuate lipid peroxidation, and thus oxidative stress, to blunt liver fibrosis, via suppression of activated HSC function, and might protect against liver tumourigenesis [99].

Leptin is secreted principally by the adipocytes of the white adipose tissue, but also by brown adipose tissue, placenta, ovaries, skeletal muscle, bone marrow and liver. It has pleitropic effects on energy homeostasis and metabolism. Leptin has anorexigenic effects decreasing appetite and increasing energy expenditure. In fact, leptin under physiological conditions prevents weight gain and positively correlates with the amount of energy stored in adipose tissue. Thus, leptin is able to activate a negative feedback loop for BMI regulation [102]. In the liver it has antilipogenic effects that are achieved by lowering the expression of sterol regulating binding protein 1 (SERBP-1). In obese patients hyperleptinemia has been described, suggesting a mechanism of leptin resistance [103]. In the setting of NASH, high to normal serum leptin levels have been found independently from BMI. Moreover, leptin is able to activate HSC, thus prompting the evolution of liver fibrosis [98]. Accordingly high serum leptin concentrations have been revealed in cirrhotic patients [104,105].

In addition leptin has important pro-inflammatory effects, exerting important effects both in innate and adaptive immunity [106,107]. Leptin can affect thymic homeostasis and the secretion of acute-phase-reactants as IL1 and TNF-α and is able to promote Th1 cell differentiation and to alter the Th1/Th2/T regs balance. In conditions of hyperleptinemia, as occurs in obesity, an expansion of the Th1 cells in the adipose tissue and an increase in pro-inflammatory cytokine secretion (as TNF-α, IL6 and IL12) have been described together with an increase of CD8+ T cells, macrophages and mast cells. These alterations are coupled with a down-regulation of the Tregs in the adipose tissue [107]. In addition leptin is implicated in the susceptibility to autoimmune and infectious diseases, given the association of increased leptin levels with chronic inflammation, autoimmune conditions and increased frequency of infections [108] and given the inhibition of autoimmunity by decreased leptin levels. Overall these data suggest a connection between nutrition, metabolism and the immune system.

Ghrelin is a gut peptide that is involved in multiple functions such as regulation of food intake, energy balance, gastric motility and growth hormone secretion. Moreover it has anti-oxidant and anti-inflammatory effects. In particular ghrelin has protective effects on the liver and reduced levels of this hormone have been found in NAFLD patients [109].

Resistin is a peptide released by adipose tissue and macrophages that is implicated in insulin resistance and has been correlated with NAFLD severity and with the development of NASH [110], probably through regulation of oxidative stress [111]. Moreover resistin has pro-inflammatory effects, as it stimulates TNF-α and IL12 secretion by macrophages through a NFκB-dependent pathway and regulates the secretion of IL6 and IL1β. Furthermore it favours liver fibrogenesis by exerting its pro-inflammatory action on HSC [97].

A recent study analysing several of these mediators in patients with NASH *versus* controls showed decreased levels of adiponectin, increased levels of leptin and resisitin and unaltered levels of ghrelin in the NASH group. Moreover an increase of the adiponectin, leptin and ghrelin levels was observed after short term antioxidant treatment [98].

Visfatin is an adipokine with insulin mimicking properties that has been described to increase the production of IL6 from human CD4+ T cells [112]. The role of this molecule in NASH, however, needs further evaluation.

## 5. Therapeutic Implications

Currently there is no approved pharmacological treatment available for NASH. The emerging role of disturbances of the immune system in the pathogenetic mechanisms of NASH opens perspectives for new potential therapeutic options through immuno-regulation [113].

Preliminary trials have used the anti-CD3 moAb, which is able to prevent induction and progression of inflammatory and autoimmune diseases.

Preclinical studies showed the efficacy of anti-CD3 moAb or of its F(ab^1^)_2_ in controlling insulin resistance in leptin deficient *ob/ob* and wild type mice. A 5-day short term treatment course appeared to be able to restore T regs in the visceral adipose tissue and to improve glucose tolerance and insulin sensitivity, despite continuation of HFD [67].

Another approach consisted of oral administration of anti-CD3 moAb paired with β-glucosylceramide (GC). Oral anti-CD3 antibody is rapidly taken up by the gut-associated lymphoid tissue (GALT) and induces CD4+CD25−latency-associated peptide (LAP)-positive Tregs, which act in a TGF-β-dependent manner. β-GC is an intermediate in the metabolic pathway of glycosphingolipids, which is able to interact with CD1d, a ligand of NTK. Treatment resulted in a decrease in pancreatic islet cell hyperplasia, fat accumulation in the liver and inflammation in adipose tissue, and was accompanied by lower blood glucose and liver enzymes. In addition an increase of CD11b+F4/80+ macrophages and TNF-α in the adipose tissue was observed [114].

A recent single-blind randomized placebo-controlled phase 2a study showed the safety of oral anti-CD3 moAb in 36 patients affected by NASH and altered glucose metabolism, including subjects with type-2 diabetes. Oral anti-CD3 moAb appeared to be safe, and was effective in reducing liver enzymes and glucose and insulin levels. Additionally, whereas blood level of CD3, CD4 and CD8 positive cells remained unchanged, a persistent increase in T reg levels was observed [115,116].

Based on promising preclinical data [117], another open-label trial tested 30-day oral administration of an anti-LPS hyperimmune bovine colostrum, Imm124-E, in 10 patients with biopsy-proven NASH and insulin resistance. Imm124-E was safe and appeared to improve insulin resistance (haemoglobin A1c (HbA1c) was significantly ameliorated together with a decrease of fasting glucose levels, improved oral glucose tolerance test, HOMA score) and lipid profile, and to alleviate related liver injury (as shown by the reduction of the liver enzymes after treatment). This improvement at the clinical level was paired with an increase in glucagon-like peptide-1 (GLP-1) and adiponectin and with an enhancement of T regs [118].

A novel drug that has been tested in NAFLD mouse models is DT56a, a compound isolated from soybean that is able to activate estrogen receptors. In leptin deficient *ob/ob* and HFD mice DT56a ameliorated glucose homeostasis (assessed by fasting blood glucose levels and glucose tolerance test), lipid profile and liver enzymes. At the immunological level, DT56a resulted in a redistribution of T regs and an increase in NKTs [119].

Cellular therapy has raised great interest in the therapeutical perspectives of NAFLD. Preclinical studies have been conducted with different subsets of cells. CD4+ Tcell transfer into obese mice reversed weight gain and insulin resistance [67], iNKT transfer decreased body fat, triglyceride levels, leptin, fatty liver and insulin sensitivity [31], while T reg expansion alone was able to reduce TNF-α-related inflammation [75] but was unable to restore overall metabolic function in obesity [77]. These treatments, however, raise feasibility concerns in the clinical setting [66] and need further development and evaluation.

Another promising therapeutical approach regards the Th17 pathway. Recently ROR-γt ligands have been developed in the treatment of autoimmune diseases. These compounds are able to inhibit the secretion of IL17 from stimulated Th17 cells, by antagonizing the activity of the nuclear receptor ROR-γt [120]. Given the important role played by the Th17 pathway in the onset and progression of metabolic and liver disease, these molecules could represent a new possible strategy in the treatment of NASH.

Finally, a recent preclinical study showed the ability of the multi-kinase inhibitor Sorafenib, already in use for the treatment of hepatocellular carcinoma, to attenuate chronic liver injury and liver fibrosis in carbon tetrachloride (CCl4)-treated mice. This antifibrogenic effect was mediated by hepatic STAT3 which in turn was activated by KC-derived IL6. These data provide evidence of the potential use of Sorafenib in the setting of advanced liver disease, characterized by fibrosis [121].

## 6. Conclusions

The pathogenesis of NASH is complex and involves different organs. The role of immune system disturbances in these multifactorial mechanisms is increasingly being recognized. Both the innate and adaptive immune system are involved, and they are disturbed at different levels. They display not only tissue specific modifications (e.g., liver and adipose tissue) but also, within the same tissue, location-specific (e.g., visceral and subcutaneous adipose tissue) discrepancies. Of note is the imbalance of the T reg/Th17 axis, which could become a target of novel therapies addressed either to enhance the T reg compartment or to suppress the Th17 pathway (e.g., inhibiting ROR-γt). Adipokines contribute to the metabolic and inflammatory features of the disease. Leptin, in particular, is involved in constituting a loop between nutrition, metabolism and the immune system.

## Figures and Tables

**Table 1:** Adipokines and other soluble mediators [97–112].

Mediator	Effect
Adiponectin	Insulin-sensitizing properties Anti-inflammatory properties Hypoadiponectinemia associated with NASH
Leptin	Pro-inflammatory properties on innate and adaptive immunity Anorexigenic effects Antilipogenic effects in the liver Insulin resistance in obesity Susceptibility to autoimmune and infectious diseases
Ghrelin	Orexigenic action Anti-oxidant effect Anti-inflammatory effect Reduced levels in NAFLD
Resistin	Favours insulin resistance Interferes with oxidative stress Stimulates cytokine release Correlation with NAFLD severity and NASH development
Visfatin	Insulin mimicking properties Stimulates IL6 secretion
